# Supplementary material for: P62/SQSTM1 binds with claudin-2 to target for selective autophagy in stressed intestinal epithelium
Source: Commun Biol. 2023 Jul 17;6:740. doi: 10.1038/s42003-023-05116-2 (PMC10352296; doi:10.1038/s42003-023-05116-2)

P62/SQSTM1 Binds with Claudin-2 to Target for Selective Autophagy in Stressed Intestinal Epithelium

*Rizwan Ahmad[1], *Balawant Kumar[1], Raju Lama Tamang[1], Geoffrey A. Talmon[2], Punita Dhawan[1,3,4] and #Amar B. Singh[1,3,4]

1. Department of Biochemistry and Molecular Biology, University of Nebraska Medical center, Omaha, USA
2. Department of Pathology, University of Nebraska Medical Center, Omaha, USA
3. Fred and Pamela Buffett Cancer Center, University of Nebraska Medical Center, Omaha, USA
4. VA Nebraska-Western Iowa Health Care System, Omaha, USA

* Denotes equal contribution

# Corresponding author

Keywords: Autophagy, Claudin-2, IBD

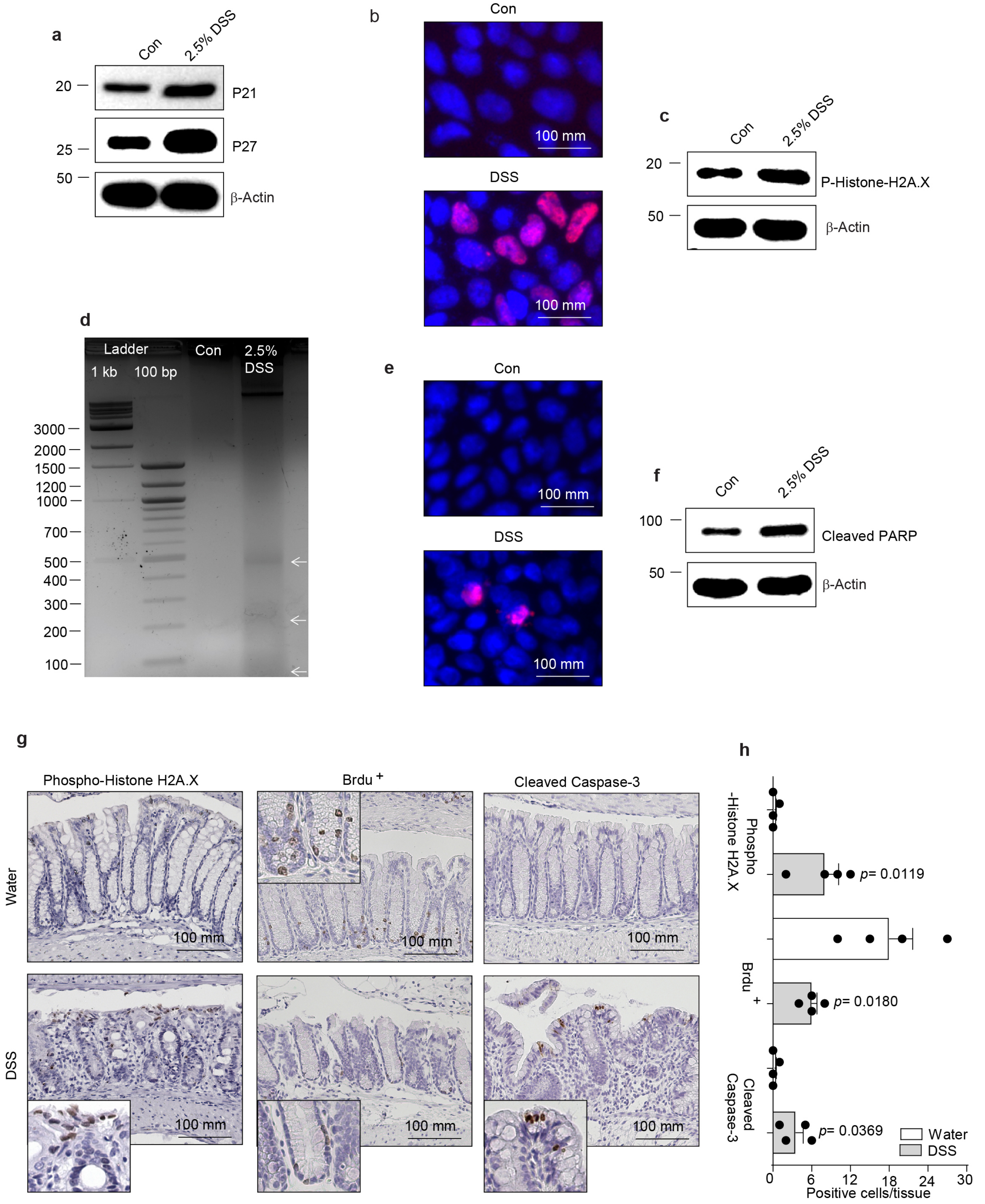

Supplementary Figure 1: *DSS-treatment induces cell cycle arrest, DNA damage and apoptosis in colonic epithelial cells.* a. Immunoblot analysis for cell-cycle inhibitor proteins, p21Cip1 and Cyclin-dependent kinase inhibitor 1B (p27Kip1) using lysates from Caco2 cells cultured in the presence of 2.5% DSS for 24 hours; b and c. Immunofluorescent and immunoblot analysis for Phospho-Histone-H2AX, a marker for double-stand DNA break; d. DNA laddering as the marker of apoptosis. Caco2 cells were treated with 2.5% DSS (w/v); e and f. Representative immunostaining for cleaved caspase-3 and immunoblotting for cleaved PARP using Caco2 cell lysates (DSS 2.5% for 24 hours in complete culture medium; g and h. immunohistochemistry and staining intensity analysis for Phospho-Histone-H2A, Brdu and cleaved caspase-3 in the colons of mice subjected to DSS (Acute) colitis. n = 4 mice/group and results are presented as means ± SEM. *p*-values from Student's t-test.

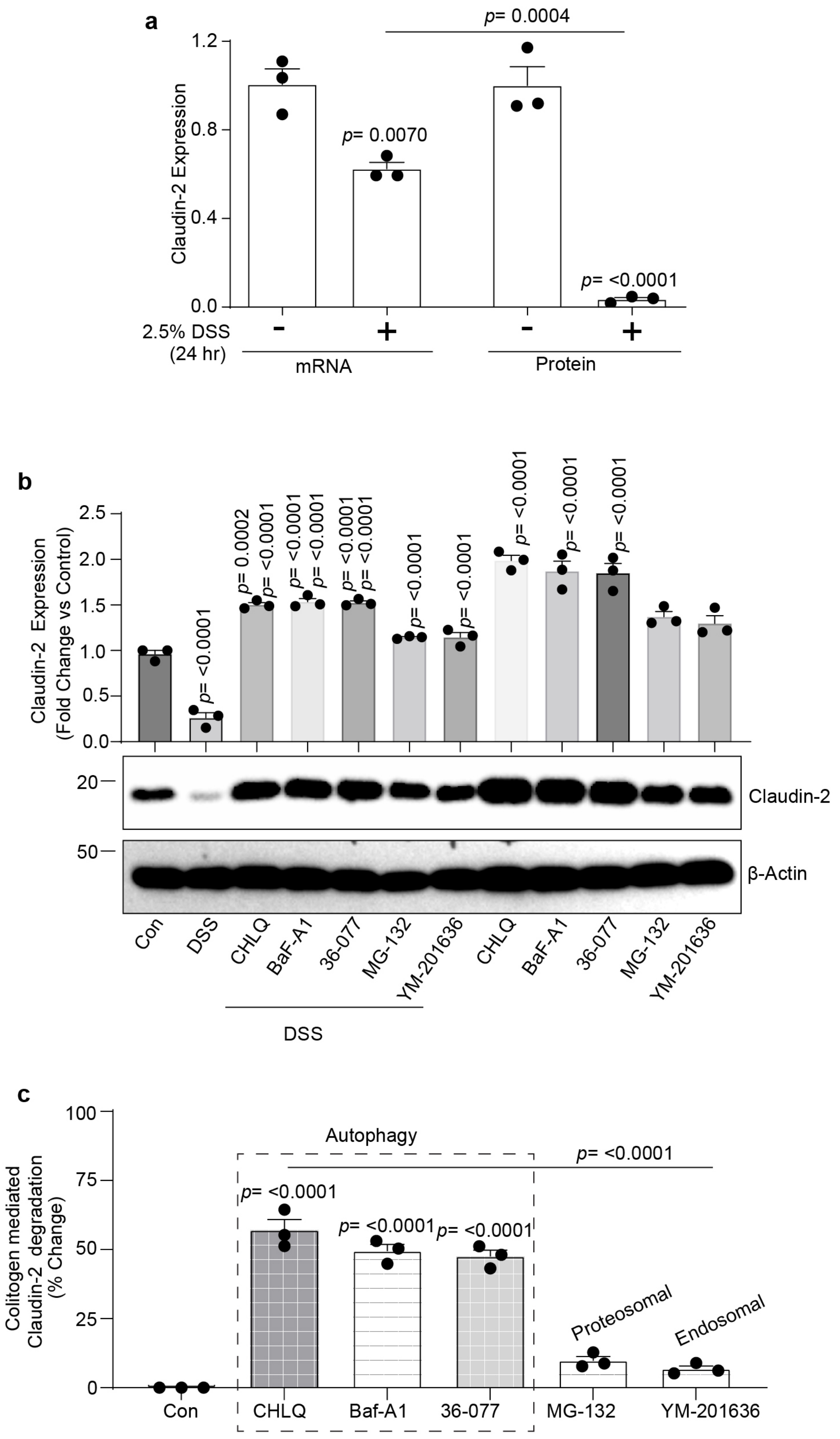

Supplementary Figure 2: *Colitogen-treatment promote autophagy and degradation of claudin-2 protein.* a. Quantification of the changes in claudin-2 mRNA and protein expression. Caco2 cells were treated with DSS for 24 hours; b and c. Immunoblotting and densitometric analysis of claudin-2 expression using cell lysates of Caco2 cells subjected to DSS (2.5%) treatment with/without chloroquine, Bafilomycin A1, VPS34 inhibitor 36-077, MG132 or YM-201636. n = 3 independent experiments. Results are presented as means ± SEM. *p*-values from one-way ANOVA followed by Tukey's post hoc test (a,b, and c).

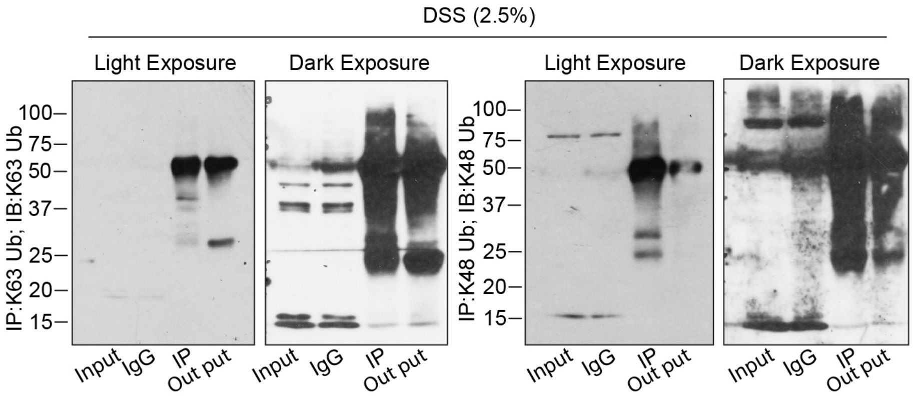

Supplementary Figure 3: *Co-immunoprecipitation analysis using anti-K63 and -K48 ubiquitin antibody.* Total cell lysate from untreated and treated with 2.5% DSS were used.

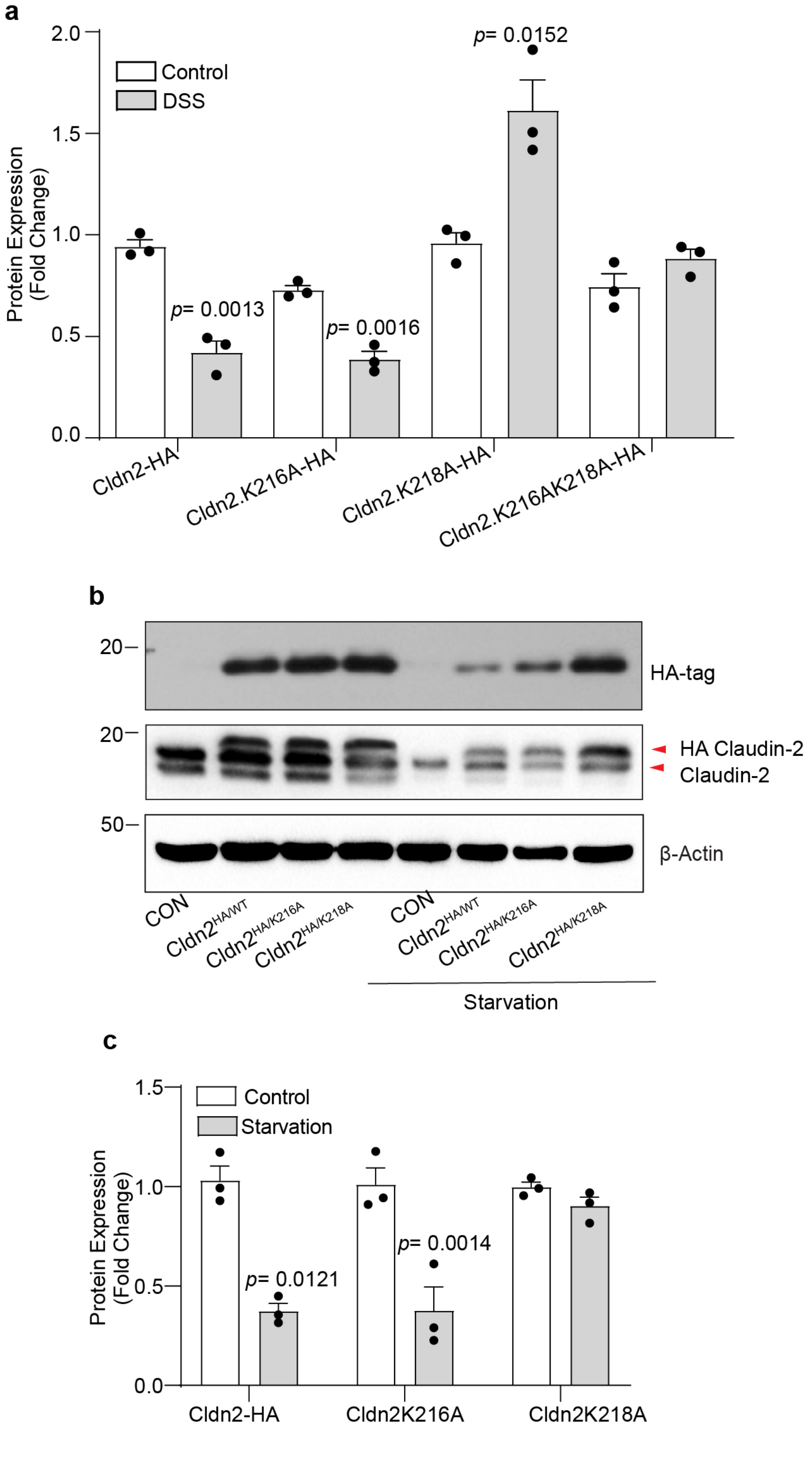

Supplementary Figure 4: *Claudin-2K218A mutant is resistant to the nutrient stress-degradation.* a. Immunoblotting using anti-HA tag antibody using total cell lysates. Caco2 cells expressing full-length or mutant claudin-2 constructs were subjected to the DSS treatment (24 hours); b-c. Immunoblotting (and densitometric analysis) using anti-claudin-2 or anti-HA-tag antibody. Cell lysate from Caco-2 cells transiently overexpressing Claudin-2-HA(Cldn2-HA), Claudin-2K216A (Cldn2K216A-HA), and Claudin-2K218A-HA (cldn2K218A-HA) constructs were used. Effect of nutrient starvation was determined versus control cells. n = 3 independent experiments and results are presented as means ± SEM. *p*-values from Student's t-test (a and c).

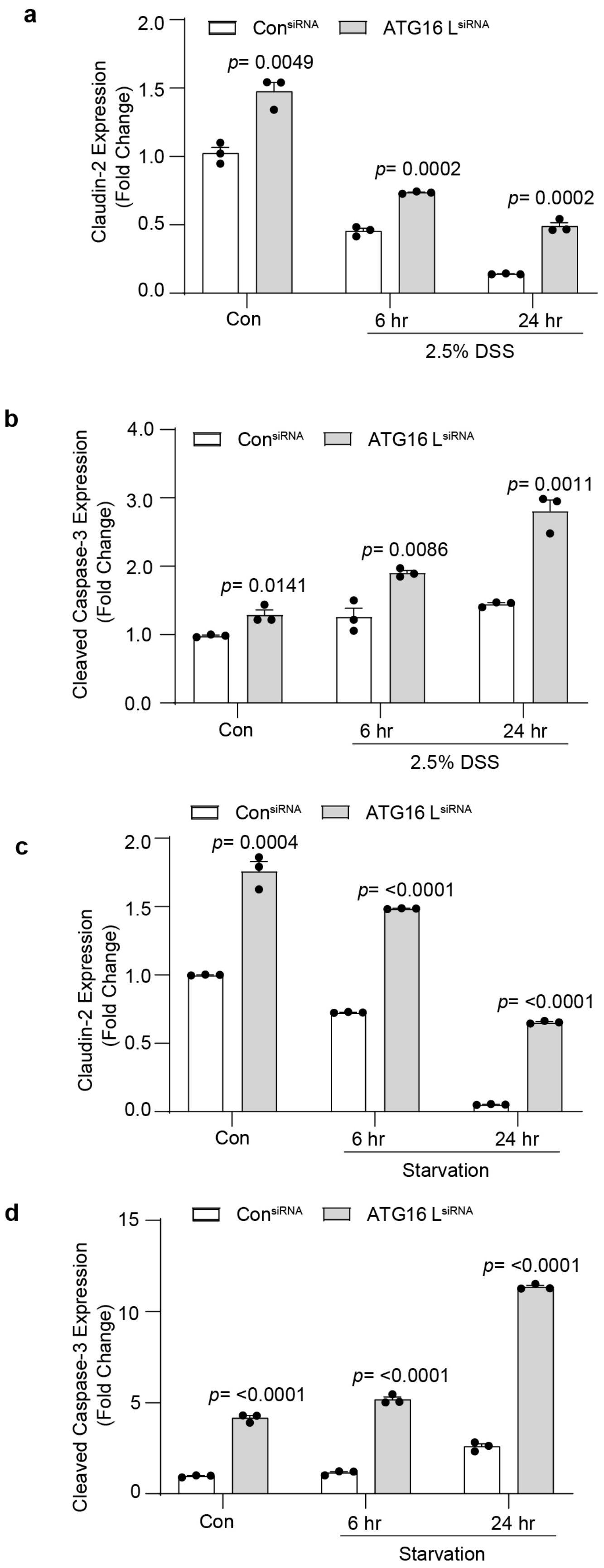

Supplementary Figure 5: *Genetic silencing of ATG16L prevents stress-induced claudin-2 degradation.* a and b. Densitometric analysis for claudin-2 and cleaved caspase-3 expression. Total cell lysates were used from Caco2 cell transiently transfected with control or ATG16L siRNA. After 24 hr of transfection, cells were exposed to 2.5% DSS in complete culture medium or (c and d) nutrient starvation for 6 and 24 hours, respectively. n = 3 independent experiments and results are presented as means ± SEM. *p*-values from Student's t-test (a,b,c, and d).

Supplementary Table 1

Predicted ubiquitination sites in claudin-2 protein

Peptide	Position	Score	Threshold
AMLLPSWKTSSYVGA	31	1.74	-1.90
VTAVGFSKGLWMECA	48	0.47	-1.90
FCQESRAKDRVAVAG	114	0.13	-1.90
PLVPDSMKFEIGEAL	157	-0.09	-1.90
PRPGQPPKVKSEFNS	216	0.13	-1.90
PGQPPKVKSEFNSYS	218	1.63	-1.90

Supplementary Table 2:

Primer sequence used for the site-directed mutagenesis.

S. N	Primer	Sequence (5′-------------------------3′)
1	CLD2.H.COM.FP	CGACTCACTATAGGGAGACCCAAGCTTATGGCCTCTCTTGGCC
2	CLDN2.RP.K216A	CCCTGTCAGGCTGTAGGAATTGAACTCACTCTTGACTGCGGG
3	CLDN2.RP.K218A	CCTGTCAGGCTGTAGGAATTGAACTCACTCGCGAC
4	CLDN2.RP.K216A. K218A	AGGCTGTAGGAATTGAACTCACTCGCGACTGCGGG
5	CLDN2.h.RP	TGGTGGTGGTGTCACACATACCTCAGGTAGGAATTG
6	CLDN2.HA.RP	CCCTCTAGATGCATGCTCGAGCTAGTGGTGGTGGTGGTGGTGTCA CACATA

Supplementary Table 3 : List of kits, and antibody

S.N.	Antibody	Company	Catalog Number
1	Claudin 2 Mouse monoclonal antibody (12H12)	Invitrogen	32-5600
2	Claudin 4 Mouse monoclonal Antibody (3E2C1)	Invitrogen	32-9400
3	E-cadherin Mouse monoclonal antibody	BD biosciences	610181
4	p27 Antibody (F-8)	Santa Cruz Biotechnology	sc-1641
5	P21 Mouse monoclonal antibody	Santa Cruz Biotechnology	Sc6246
6	HA-Tag Rabbit monoclonal antibody	Cell signaling Technology	2367S
7	K63-linkage Specific Polyubiquitin	Cell signaling Technology	5621S
8	K48-linkage Specific Polyubiquitin	Cell signaling Technology	12805S
9	ATG16 Antibody	ProteinTech	29445-1-AP
10	β-actin Antibody	Sigma	A5316-100UL
11	Recombinant Anti-Ubiquitin (linkage specific K63) antibody	Abcam	ab179434
12	jetPRIME transfection Reagent	Polyplus transfection	114-07
13	VECTASTAIN Elite ABC Universal PLUS Kit	Vector Laboratory	PK-8200
14	Claudin-2 siRNA	Thermo fisher	4392420 (assay ID s225076)
15	Corning Matrigel Matrix	Corning	356255, 354236
16	LAMP1 (D2D11) Monoclonal Rabbit	Cell signaling Technology	9091
17	LC3B monoclonal antibody	Sigma	L7543
18	p62/SQSTM1 monoclonal antibody	Sigma	L0076
19	Anti-human P62 siRNA	Invitrogen	s16962
20	ATG16L1 siRNA	Invitrogen	s30070
21	Ubiquitin (E4I2J)	Cell signaling Technology	43124
22	Claudin2 shRNA	Sigma	TRCN0000091500
23	Duolink In Situ Red Starter Kit Mouse/Rabbit	Sigma	DUO92101-1KT
24	Cleaved Caspase 3 antibody	Cell signaling Technology	9661S
25	Cleaved PARP antibody	Cell signaling Technology	5625S
26	Phospho-Histone H2A.X (Ser139) (20E3)	Cell signaling Technology	9718

Supplementary Fig. 6

Figure-1a

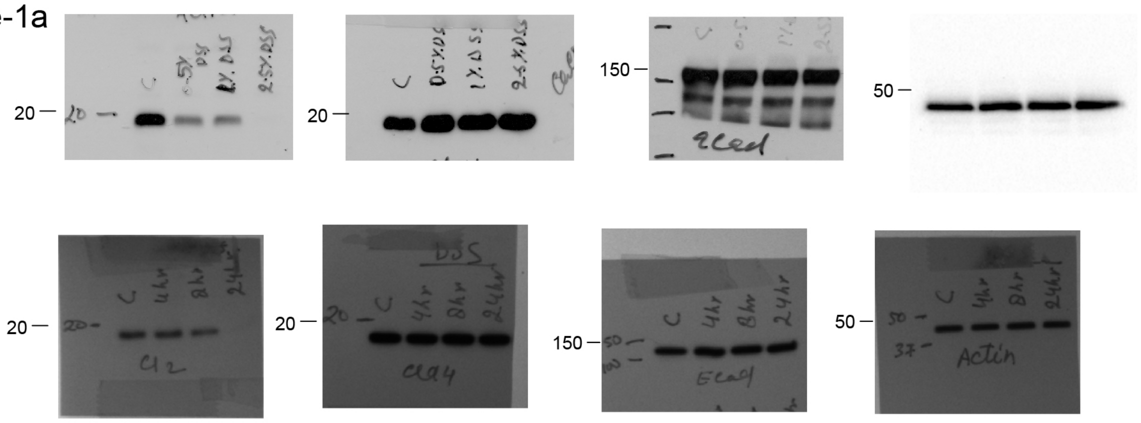

Figure-1b

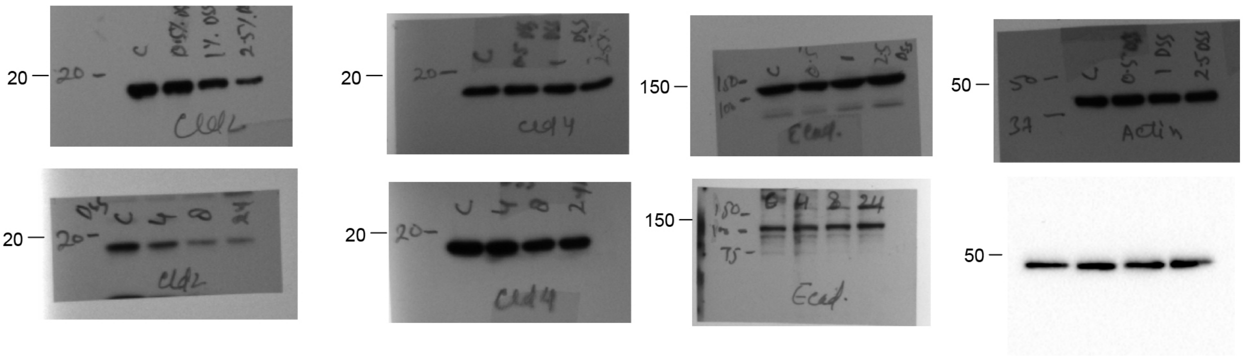

Figure-1c

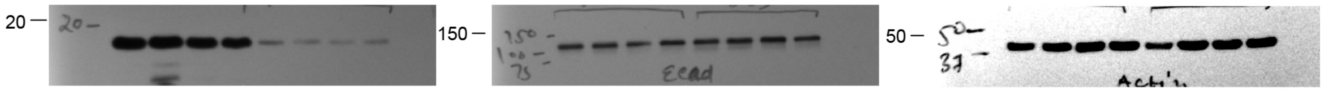

Figure-1d

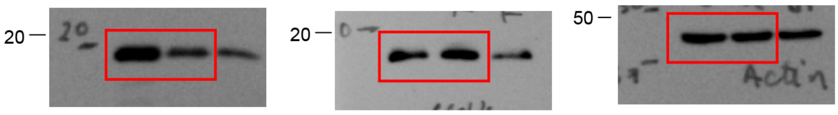

Figure-1e

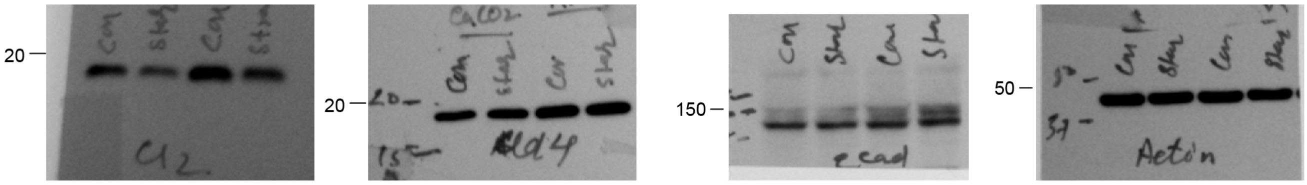

Supplementary Fig. 6 (continued)

Figure-2a

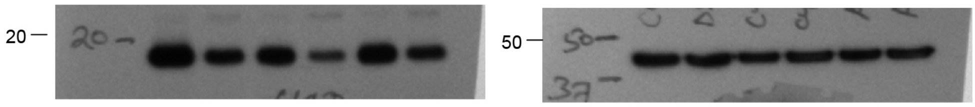

Figure-2b

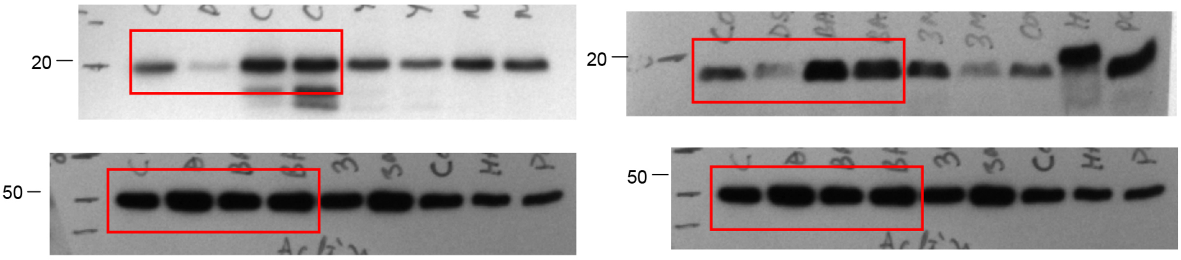

Figure-2c

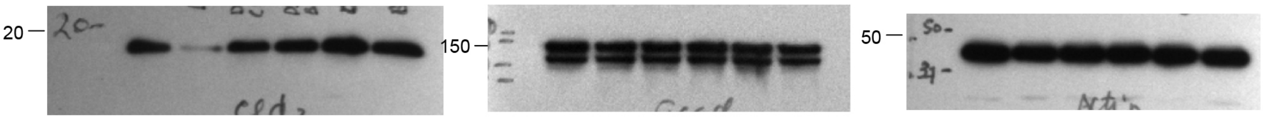

Figure-2g

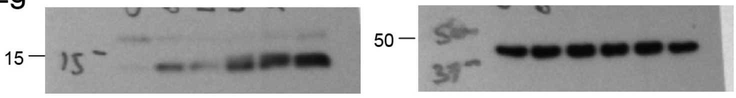

Figure-2h

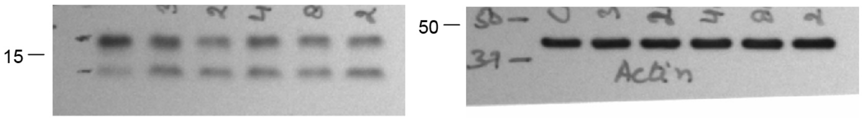

Figure-2i

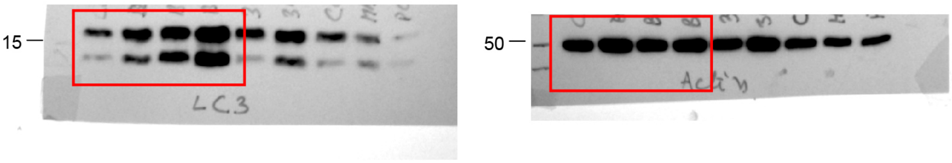

Figure-2j

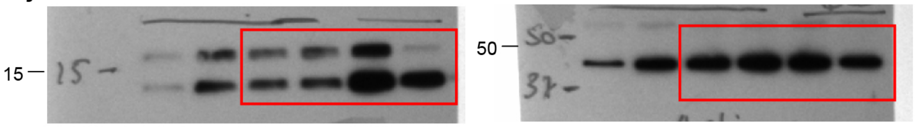

Supplementary Fig. 6 (continued)

Figure-3c

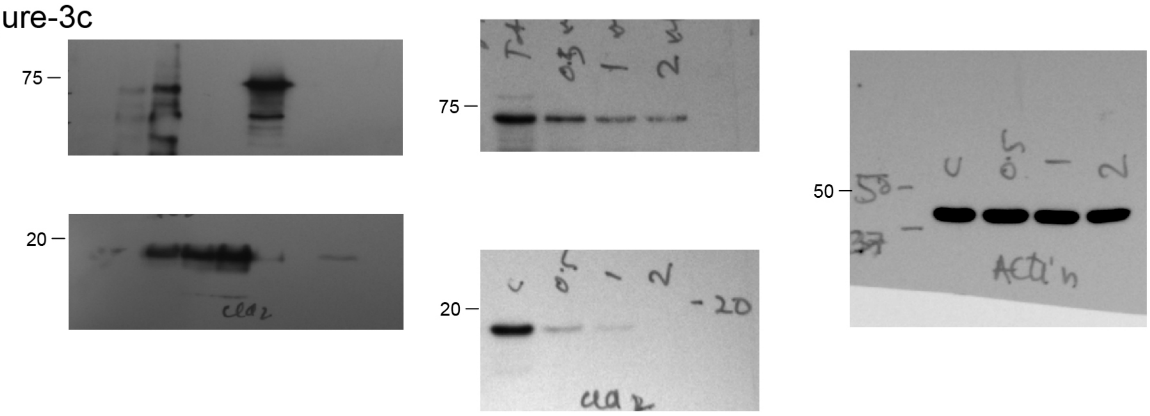

Figure-3d

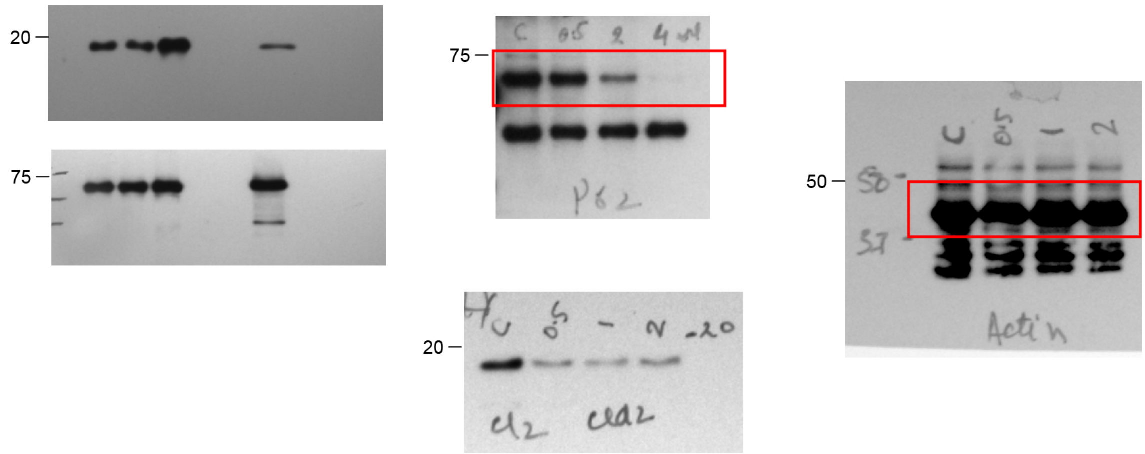

Figure-3e

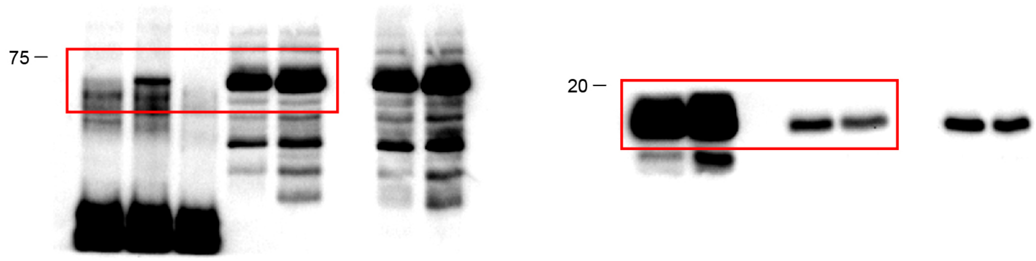

Figure-3g

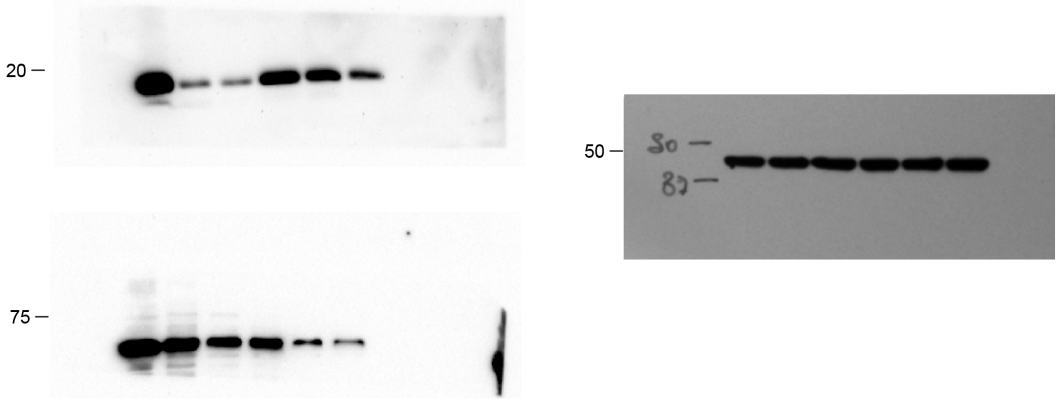

Supplementary Fig. 6 (continued)

Figure-4a

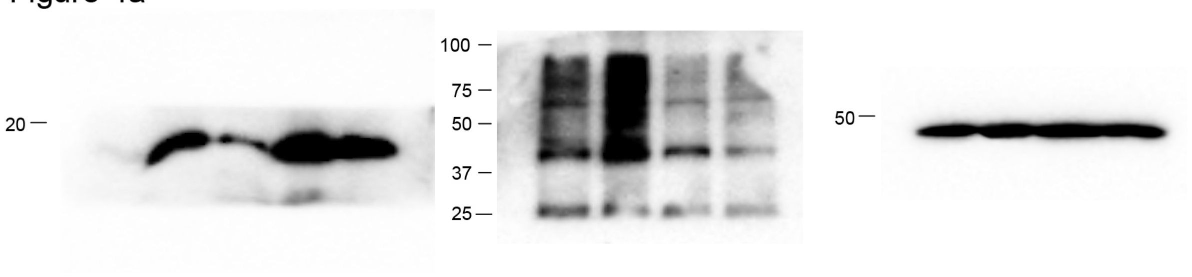

Figure-4b

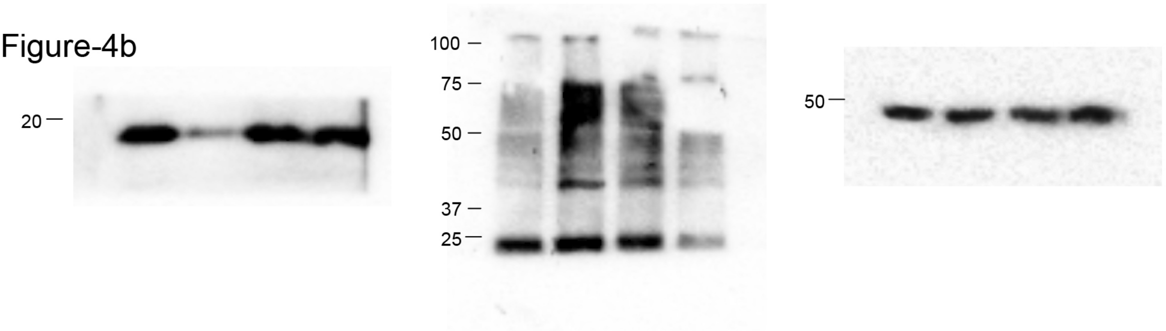

Figure-4c

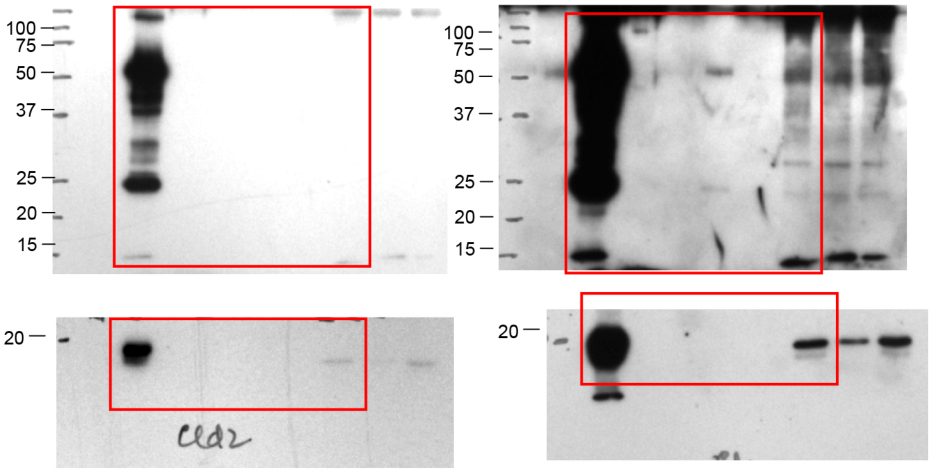

Figure-4e

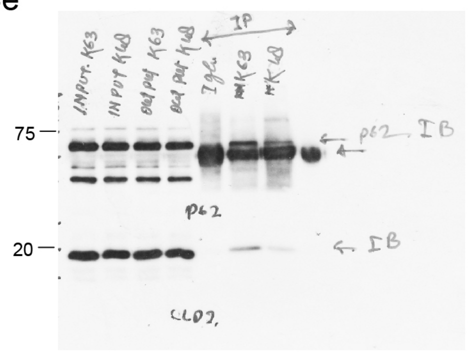

Supplementary Fig. 6 (continued)

Figure-5b

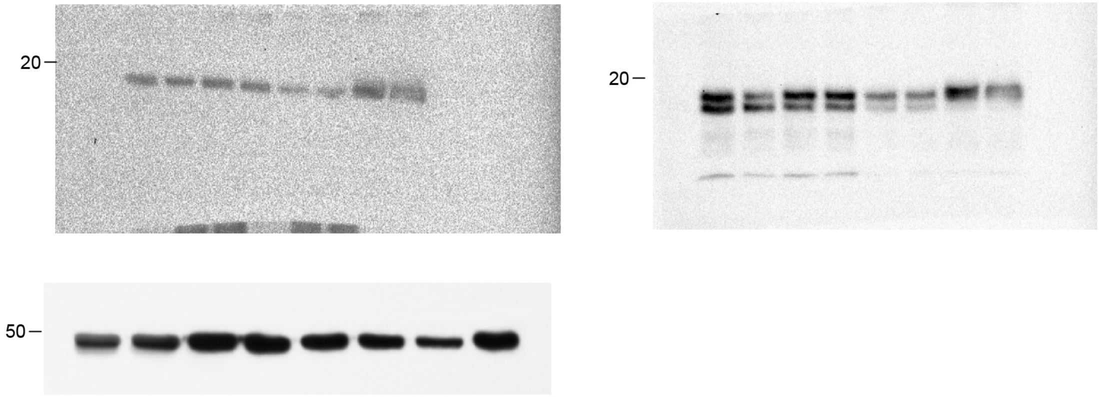

Figure-5c

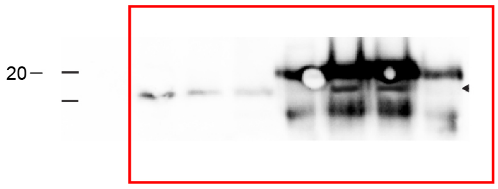

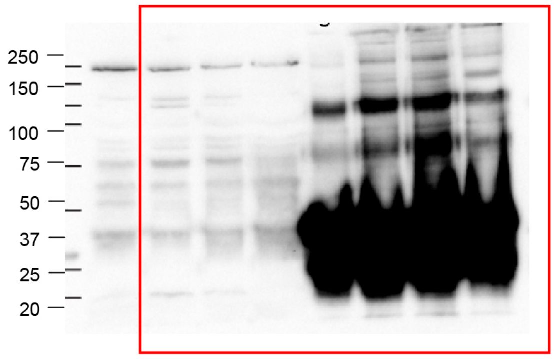

Figure-5d

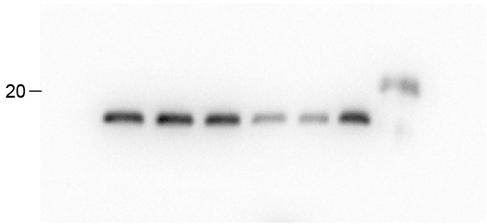

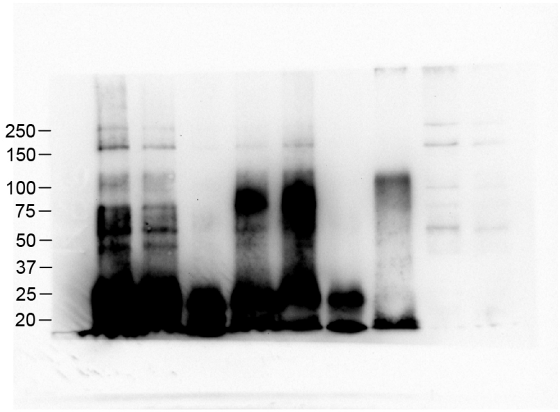

Supplementary Fig. 6 (continued)

Figure-6b

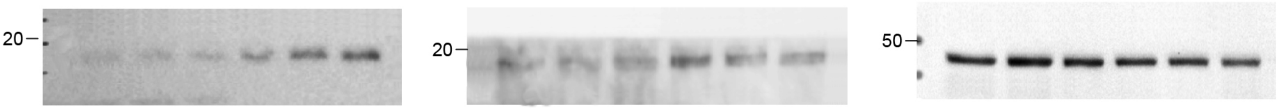

Figure-6c

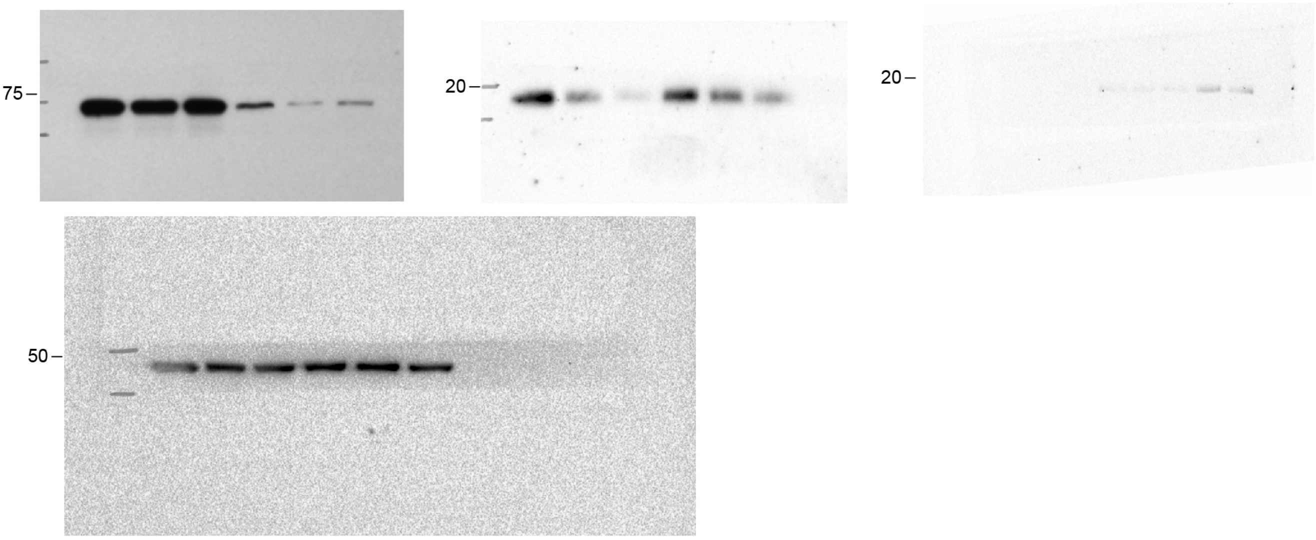

Figure-6d

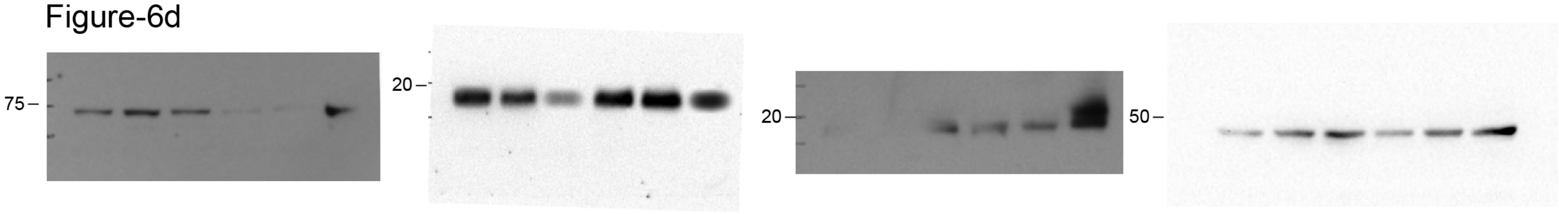

Figure-6f

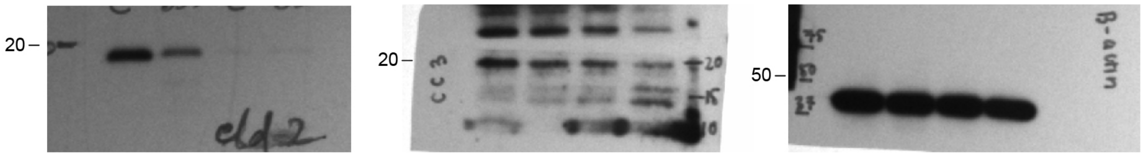

Figure-6h

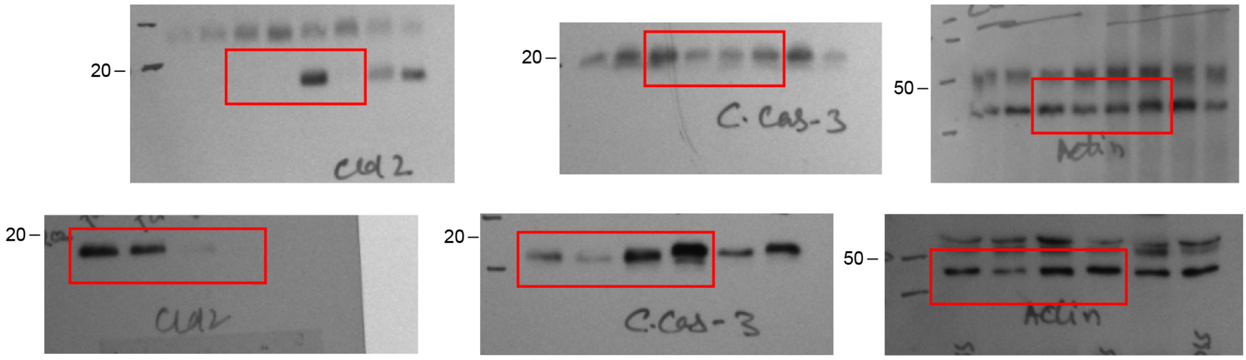

Figure-6j

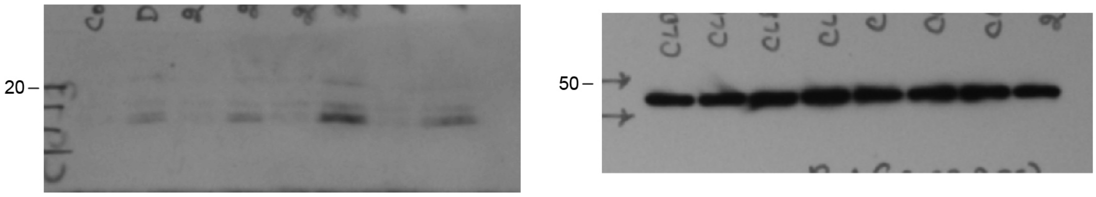

Supplementary Fig. 6 (continued)

Supp. Figure-1a

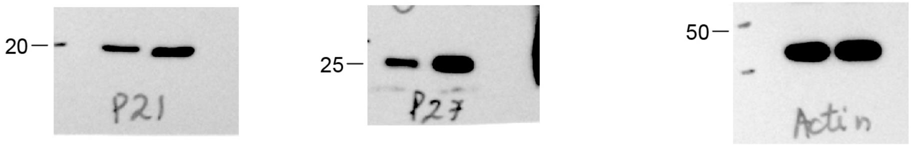

Supp. Figure-1c

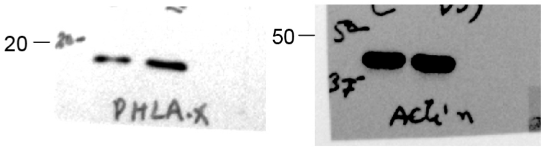

Supp. Figure-1d

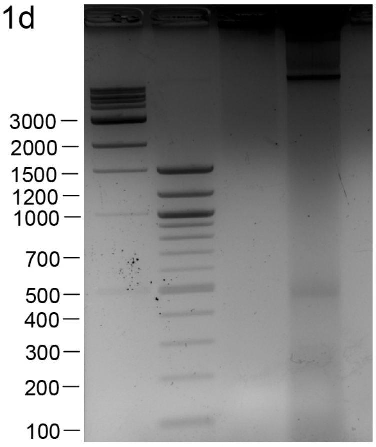

Supp. Figure-1f

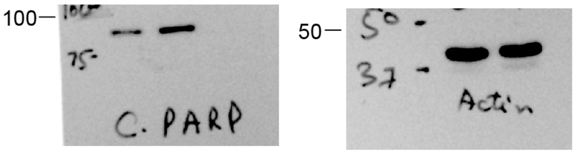

Supplementary Fig. 6 (continued)

Supp. Figure-2b

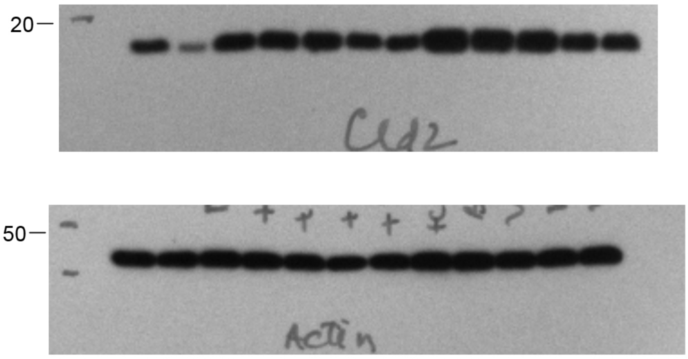

Supplementary Fig. 6 (continued)

Supp. Figure-3

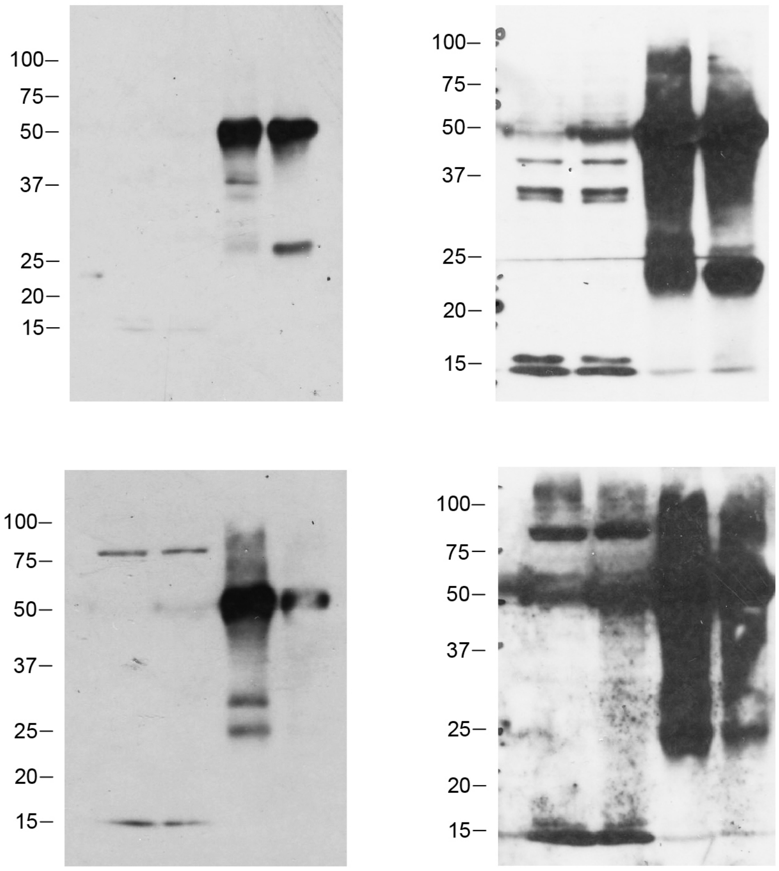

Supplementary Fig. 6 (continued)

Supp. Figure-4b

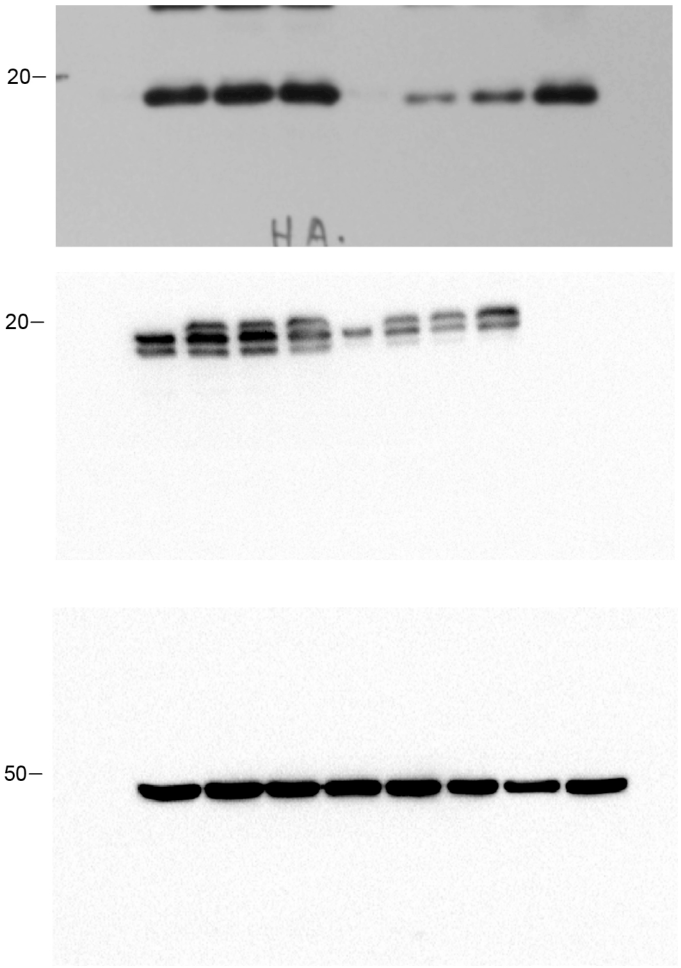

Supplement: Supplementary file 2 — Supplementary information [file 42003_2023_5116_MOESM2_ESM.pdf]
