# Supplementary material for: P62/SQSTM1 binds with claudin-2 to target for selective autophagy in stressed intestinal epithelium
Source: Commun Biol. 2023 Jul 17;6:740. doi: 10.1038/s42003-023-05116-2 (PMC10352296; doi:10.1038/s42003-023-05116-2)
Supplement: Supplementary file 5 — Description of Additional Supplementary Files [file 42003_2023_5116_MOESM5_ESM.pdf]

Description of Additional Supplementary Files

File name: Supplementary Data 1
Description: Source of all graph data for main figures.

File name: Supplementary Data 2
Description: Source of all graph data for supplementary figures.
