# Supplementary material for: P62/SQSTM1 binds with claudin-2 to target for selective autophagy in stressed intestinal epithelium
Source: Commun Biol. 2023 Jul 17;6:740. doi: 10.1038/s42003-023-05116-2 (PMC10352296; doi:10.1038/s42003-023-05116-2)
Supplement: Supplementary file 6 — Reporting Summary [file 42003_2023_5116_MOESM6_ESM.pdf]

nature portfolio

Corresponding author(s): Amar B Singh

Last updated by author(s): Jun 26, 2023

Reporting Summary

Nature Portfolio wishes to improve the reproducibility of the work that we publish. This form provides structure for consistency and transparency in reporting. For further information on Nature Portfolio policies, see our Editorial Policies and the Editorial Policy Checklist.

Statistics

For all statistical analyses, confirm that the following items are present in the figure legend, table legend, main text, or Methods section.

n/a	Confirmed	
☐	☒	The exact sample size (*n*) for each experimental group/condition, given as a discrete number and unit of measurement
☐	☒	A statement on whether measurements were taken from distinct samples or whether the same sample was measured repeatedly
☐	☒	The statistical test(s) used AND whether they are one- or two-sided<br>*Only common tests should be described solely by name; describe more complex techniques in the Methods section.*
☒	☐	A description of all covariates tested
☒	☐	A description of any assumptions or corrections, such as tests of normality and adjustment for multiple comparisons
☐	☒	A full description of the statistical parameters including central tendency (e.g. means) or other basic estimates (e.g. regression coefficient) AND variation (e.g. standard deviation) or associated estimates of uncertainty (e.g. confidence intervals)
☒	☐	For null hypothesis testing, the test statistic (e.g. *F*, *t*, *r*) with confidence intervals, effect sizes, degrees of freedom and *P* value noted<br>*Give P values as exact values whenever suitable.*
☒	☐	For Bayesian analysis, information on the choice of priors and Markov chain Monte Carlo settings
☒	☐	For hierarchical and complex designs, identification of the appropriate level for tests and full reporting of outcomes
☒	☐	Estimates of effect sizes (e.g. Cohen's *d*, Pearson's *r*), indicating how they were calculated

Our web collection on statistics for biologists contains articles on many of the points above.

Software and code

Policy information about availability of computer code

Data collection	it was not required
Data analysis	Used an online software (http://bdmpub.biocuckoo. org/prediction.php) for the identification for probable lysine sites for ubiquitination in claudin-2 protein and Prism 9 (GraphPad)

For manuscripts utilizing custom algorithms or software that are central to the research but not yet described in published literature, software must be made available to editors and reviewers. We strongly encourage code deposition in a community repository (e.g. GitHub). See the Nature Portfolio guidelines for submitting code & software for further information.

Data

Policy information about availability of data

All manuscripts must include a data availability statement. This statement should provide the following information, where applicable:

- Accession codes, unique identifiers, or web links for publicly available datasets
- A description of any restrictions on data availability
- For clinical datasets or third party data, please ensure that the statement adheres to our policy

it was not required

Human research participants

Policy information about studies involving human research participants and Sex and Gender in Research.

Reporting on sex and gender	N/A
Population characteristics	N/A
Recruitment	N/A
Ethics oversight	N/A

Note that full information on the approval of the study protocol must also be provided in the manuscript.

Field-specific reporting

Please select the one below that is the best fit for your research. If you are not sure, read the appropriate sections before making your selection.

[x] Life sciences [ ] Behavioural & social sciences [ ] Ecological, evolutionary & environmental sciences

For a reference copy of the document with all sections, see nature.com/documents/nr-reporting-summary-flat.pdf

Life sciences study design

All studies must disclose on these points even when the disclosure is negative.

Sample size	Sample size was based on our and other researcher's previous experiences. No formal power calculations were performed.
Data exclusions	Data was not excluded from any of the analyses.
Replication	the n in each experiment is shown in respective figure legends and manuscripts text.
Randomization	Animals were randomized on the basis of the body weight and age.
Blinding	The analysis was being performed on control and treatment groups.

Reporting for specific materials, systems and methods

We require information from authors about some types of materials, experimental systems and methods used in many studies. Here, indicate whether each material, system or method listed is relevant to your study. If you are not sure if a list item applies to your research, read the appropriate section before selecting a response.

Materials & experimental systems

n/a	Involved in the study	
[ ]	[x]	Antibodies
[ ]	[x]	Eukaryotic cell lines
[x]	[ ]	Palaeontology and archaeology
[ ]	[x]	Animals and other organisms
[x]	[ ]	Clinical data
[x]	[ ]	Dual use research of concern

Methods

n/a	Involved in the study	
[x]	[ ]	ChIP-seq
[x]	[ ]	Flow cytometry
[x]	[ ]	MRI-based neuroimaging

Antibodies

Antibodies used	Claudin 2 (32-5600, Thermo Fisher Scientific), Claudin 4 (32-9400,Thermo Fisher Scientific),E-cadherin (610181,BD),P27 (sc-1641, santacrutz), P21 (Sc6246, santacrutz), HA-Tag (2367S,CST),K63-linkage Specific Polyubiquitin (5621S, ab179434,CST, Abcam), K48-linkage Specific Polyubiquitin (12805S, CST), ATG16 (29445-1-AP< proteintech), LAMP1 (9091,CST), LC3B (L7543, Sigma), p62/SQSTM1 (L0076, Sigma), Cleaved Caspase 3 (9661S,CST), Cleaved PARP antibody (5625S,CST), Phospho-Histone H2A.X (9718, CST), Ubiquitin (43124 ,CST)
Validation	validation could be found at manufacturer's website

Eukaryotic cell lines

Policy information about cell lines and Sex and Gender in Research

Cell line source(s)	ATCC
Authentication	Yes
Mycoplasma contamination	Mycoplasma free cells were used.
Commonly misidentified lines (See ICLAC register)	None

Animals and other research organisms

Policy information about studies involving animals; ARRIVE guidelines recommended for reporting animal research, and Sex and Gender in Research

Laboratory animals	C57BL/6 genetic mouse line were used.
Wild animals	N/A
Reporting on sex	N/A
Field-collected samples	N/A
Ethics oversight	All experiments were conducted with protocols approved by the University of Nebraska Medical Center Institutional Animal Care and Use Committee.

Note that full information on the approval of the study protocol must also be provided in the manuscript.
